# USP11 degrades KLF4 via its deubiquitinase activity in liver diseases

**DOI:** 10.1111/jcmm.16709

**Published:** 2021-06-10

**Authors:** Heeyoung Yang, Daeui Park, Jeongho Ryu, Tamina Park

**Affiliations:** ^1^ Department of Predictive Toxicology Korea Institute of Toxicology Daejeon Korea; ^2^ Department of Human and Environmental Toxicology University of Science and Technology Daejeon Korea

**Keywords:** hepatic steatosis, hepatocellular carcinoma, KLF4, ubiquitination, USP11

## Abstract

Krüppel‐like factor 4 (KLF4) is a zinc‐finger containing DNA‐binding transcription factor involved in tumorigenesis and acts as a tumour suppressor or an oncogene depending on the tissue. In hepatocellular carcinoma (HCC), KLF4 has been considered as a tumour suppressor, although the mechanism underlying its action remains largely unknown. In this study, we identified the ubiquitin‐specific peptidase USP11 as a KLF4‐interacting deubiquitinating enzyme using a proteomic approach. USP11 destabilizes KLF4 through the removal of K63‐dependent polyubiquitination, thereby inhibiting KLF4 expression. We also provide mechanistic insights into KLF4 degradation and show that USP11 depletion inhibits growth and chemoresistance of HCC cells by enhancing KLF4 stability. Importantly, lipid content was reduced and genes involved in fatty acid metabolism were down‐regulated in an in vitro steatosis conditions upon USP11 knockout. Finally, elevated USP11 and reduced KLF4 levels were detected both in a hepatic steatosis in vitro model and in public clinical data of non‐alcoholic fatty liver disease and HCC patients. Collectively, these findings suggest that USP11, as KLF4‐binding partner, is an important mediator of hepatic tumorigenesis that functions via degradation of KLF4 and is a potential treatment target for liver diseases.

## 1 INTRODUCTION

Krüppel‐like factors (KLFs) are zinc‐finger containing DNA‐binding transcription factors of which 18 family members are ubiquitously expressed in diverse cell types. Due to their broad involvement in cellular processes, such as differentiation, proliferation, migration and stem cell reprogramming, a better understanding of KLFs’ role in human diseases is of great interest.^1^, ^2^ KLF expression has been shown to be altered in human cancer, as they can act as tumour suppressors or oncogenes, depending on the cellular context and the targeted substrate, to regulate cancer cell proliferation, apoptosis and metastasis.^3^, ^4^ Among the KLF family, KLF4 is a key regulator of normal cell differentiation and is also one of four key factors implicated in pluripotent stem cell induction.^5^ KLF4 may have conflicting roles in tumorigenesis in tissue type–dependent manner, switching its role from anti‐apoptotic to pro‐apoptotic under certain conditions. KLF4 prevents tumorigenesis in colon, bladder, lung, gastric, intestinal and prostate cancers, as well as leukaemia and neuroblastoma, whereas it promotes the development of breast, skin, and head and neck cancers.^4^, ^6^, ^7^ KLF4 is commonly known as a suppressor of cell cycle progression as it induces the expression of the cell cycle inhibitor p21^Cip1/Waf1^ and inhibits the cell cycle promoting genes, *CCND1* and *CCNB1*.^8^, ^9^ Additionally, KLF4 acts as an anti‐apoptotic transcription factor by suppressing the p53‐dependent apoptotic pathway, in particular, through inhibition of TP53 and BAX expression.^10^ Overall, KLF4 is a key player in numerous physiological and pathological processes.

In hepatocellular carcinoma (HCC), the loss of KLF4 expression is closely correlated with cancer progression and reduced overall survival.^11^, ^12^ KLF4 can regulate HCC differentiation and progression, and it inhibits HCC cell migration and invasion, mediated by the hepatocyte nuclear factor, HNF6.^13^ KLF4 also inhibits the oncogenic TGFβ signals via induction of Smad7.^14^ These findings suggest that KLF4 could be a potential therapeutic target for liver diseases, such as fibrosis and cirrhosis, and prevent their subsequent progression to HCC. However, the underlying molecular mechanisms for modulating KLF4 expression in HCC remain poorly understood. Given KLF4 involvement in cell fate decision in tumorigenesis, its activity is critically regulated both transcriptionally and post‐transcriptionally through methylation, acetylation, phosphorylation, ubiquitination and sumoylation, in a context‐dependent manner.^3^ KLF4 has a short half‐life and turnover rate, meaning that it is regulated post‐translationally via ubiquitination.^15^ A recent study revealed different elements of the ubiquitination process that are involved in the regulation of KLF4 protein stability in various cell types and cancers. In case of E3 ligases, for example Von Hippel‐Lindau (pVHL) is involved in breast and colon cancer, FBXO32 in breast cancer, Cdh1‐anaphase‐promoting complex in lung cells, β‐TrCP in stem cell self‐renewal and TRAF7 in HCC.^16^, ^17^, ^18^, ^19^ In contrast, deubiquitinating enzymes (DUBs) can reversibly cleave ubiquitin(s) off‐target proteins and rescue substrates from post‐translational modification.^20^ This group of enzymes contains about ~100 DUBs that are classified into six subfamilies based on sequence and domain conservation. Although DUBs are important regulatory elements of many biological processes, including protein turnover and ubiquitin recycling into monomers, the detailed mechanism of KLF4 regulation by DUBs remains unknown.

USP11 is one of the most common DUBs and belongs to the ubiquitin‐specific processing protease (USP) family. It is involved in multiple signalling cascades including TGFβ, p21, p53, NF‐βB and Notch signals, ultimately regulating the stability of their downstream substrates.^21^, ^22^, ^23^ USP11 has been recognized as a tumour suppressor in lung cancer through the regulation of Mgl‐1.^24^ In contrast, USP11 is highly associated with tumorigenesis in other cancer types such as breast, ovarian, colorectal, pancreatic cancer, melanoma, glioma and squamous cell carcinoma, due to its effect on different signalling pathways.^25^, ^26^, ^27^ Recently, USP11 was shown to promote HCC development,^28^ but the underlying molecular mechanisms involved in this pathogenic process remain poorly understood.

In this study, we used a proteomic approach to identify KLF4‐interacting DUBs and firstly discovered that USP11 was responsible for deubiquitinating KLF4 in HCC cells. USP11 deubiquitinates K63‐dependent polyubiquitination of KLF4 and suppresses its stability. Using HCC cells engineered to lack USP11, we clarified that loss of USP11 restrains HCC tumorigenesis by promoting KLF4 accumulation. More importantly, we demonstrated the negative correlation between KLF4 and USP11 expression in liver diseases such as non‐alcoholic fatty liver disease (NAFLD) and HCC. Collectively, these findings add further evidence that USP11 is a major regulator of HCC progression via direct regulation of KLF4 expression.

## 2 MATERIALS AND METHODS

### 2.1 Cells, antibodies and reagents

Human embryonic kidney (HEK) 293, HepG2, Hep3B, Sk‐Hep1 and THLE2 were obtained from American Type Culture Collection (ATCC). Huh7 and SNU423 were purchased from Korea Cell Line Banks (KCLB). According to the manufacturer's instructions, the growth culture medium was prepared by mixing DMEM or EMEM with 10% FBS and additives (Gibco). For the cultivation of THLE2 cells, the BEGM Bullet Kit (CC‐3170) from Lonza was used. The Bullet Kit contains BEBM basal medium and supplements. The final growth medium consists of the following: BEBM supplemented with 10% FCS, bovine pituitary gland extract, hydrocortisone, epidermal growth factor (EGF), insulin, triiodothyronine, transferrin, retinoic acid, 5 ng/mL human recombinant EGF (Gibco) and 70 ng/mL ο‐phosphorylethanolamine (Sigma‐Aldrich). All cells applied in this study were cultured at 37℃ in a humidified 5% CO_2_ atmosphere. Polyclonal antibodies against the epitope tags (HA and Myc), KLF4, USP11, ubiquitin and β‐actin were obtained from Santa Cruz Biotechnology, Inc Anti‐Flag antibody, anti‐Flag‐M2 affinity gel, cycloheximide (CHX), sorafenib, palmitic and oleic acid were purchased from Sigma‐Aldrich. Lipid contents of cells were measured with commercial Triglyceride assay kit (Abcam).

### 2.2 Isolation of KLF4 interactors with a proteomic approach

For identifying KLF4 interactome,^29^ HepG2 cells were transfected with Flag‐tagged KLF4 expression plasmids. The transfected cells were lysed with 1 × Nonidet P‐40 lysis buffer and pre‐cleaned by protein A/G agarose beads. Flag‐tagged KLF4 proteins were immunoprecipitated with anti‐Flag Abs beads, and the immune complex was eluted from the agarose with 100 μmol/L Flag peptide (Sigma‐Aldrich). The eluted proteins were digested with trypsin and characterized by mass spectrometry.

### 2.3 Plasmids and lentivirus transduction

Full‐length KLF4 or USP11 was PCR‐amplified from human cDNA and sub‐cloned to p3xFlag‐CMV10 or pCMV‐Myc from Addgene. Various deletion mutants were generated using PCR. Catalytically inactive USP11 (C318S) was generated by using DpnI‐mediated site‐directed mutagenesis (Qiagen). To stably knock‐down endogenous USP11 expression, shRNA sequence for USP11 was cloned into pLKO.1‐TRC cloning plasmid (Addgene). pLKO.1‐TRC control or USP11 shRNA expression plasmid was transfected to HEK293 cells with lentiviral packaging shRNA expression vector (pMD2.G and psPAX2, Addgene) using Lipofectamin 2000. Medium containing lentivirus was collected 48 hours later and concentrated. Target cells were infected with lentivirus‐containing supernatant and polybrene (8 μ g/mL) for 48 hours according to the manufacturer's instructions. Infected cells were selected with puromycin (2 μ g/mL for HCC cells, Santa Cruz Biotechnology) after infection. Stably transfected cells were maintained with regular medium for further analysis. The used shRNA sequences against human USP11 are as follows:

sense: CCGGCCGTGACTACAACAACTCCTACTCGAGTAGGAGTTGTTGTAGTCACGGTTTTTG,

anti‐Sense: AATTCAAAAACCGTGACTACAACAACTCCTACTCGAGTAGGAGTTGTTGTAGTCACGG.

### 2.4 Cell transfection and co‐immunoprecipitation assay

Transient transfection was performed using Lipofectamine 2000 (Invitrogen) according to the manufacturer's instruction. 2 days after transfection, cells were lysed in 1 × Nonidet P‐40 lysis buffer and freshly added protease inhibitor cocktail. After being pre‐cleared with protein A/G agarose beads, the lysates were mixed with indicated antibodies (1 μ g) for overnight, followed by the addition of protein A/G plus‐agarose beads (Santa Cruz) for the additional 2 hours at 4℃ with gentle shaking. Immunoprecipitated proteins were washed out four times with lysis buffer and boiled in 2 × SDS sample buffer and subjected to Western blot analysis.

### 2.5 In vivo and in vitro deubiquitylation assay

For KLF4 deubiquitylation in vivo,^30^ transfected cells with various combinations of plasmids DNA were lysed with NP‐40 lysis buffer. Cell lysates were immunoprecipitated with KLF4 antibodies or Flag‐M2 beads and analysed by Western blotting anti‐HA or anti‐Ub antibodies. For in vitro deubiquitylation experiments,^29^ Flag‐KLF4 was transfected together with HA‐Ub in HEK293 cells. Ubiquitinated KLF4 proteins were enriched by Flag‐M2 beads and eluted with the Flag peptide. The purified ubiquitinated KLF4 proteins were incubated with GST‐USP11 proteins (Abcam) in deubiquitylation buffer (50 mmol/L Tris‐HCL, pH 8.0, 50 mmol/L NaCl_,_ 1 mmol/L EDTA, 1 mmol/L DTT and 5% glycerol) at 37℃ for 2 hours. KLF4 ubiquitination was detected by Western blotting with anti‐HA antibodies.

### 2.6 Western blotting

Protein samples were boiled and separated on 8% SDS‐PAGE gels followed by electro‐transferring to PVDF membranes (Bio‐Rad Laboratories). After blocking with 5% non‐fat milk in Tris‐buffered saline containing 0.1% Tween‐20 for 1 hour, the membranes were incubated with specific primary antibodies overnight at 4℃. Finally, antibody‐bound proteins were detected by chemiluminescence (Bio‐Rad). When necessary, membranes were stripped by incubation in stripping buffer (Thermo Fisher), washed and then reprobed with other antibodies as indicated.

### 2.7 Immunofluorescence staining

For detection of subcellular localization by immunofluorescence, cells were cultured in iDIBI chamber for 24 hours, washed with PBS and fixed with 4% paraformaldehyde for 15 minutes. The fixed cells were then washed with PBS, permeabilized for 10 minutes with 0.1% Triton X‐100, blocked for 1h in 5% BSA and incubated with the antibodies: anti‐KLF4 (1:200) and anti‐USP11 (1:200) for overnight at 4℃. The nuclei were stained with DAPI (Abcam), and images were visualized with fluorescence microscopy (Olympus).

### 2.8 Real‐time quantitative RT‐PCR

Total RNA from cells was isolated with RNeasy Mini Kit and was reverse transcribed with GoScript reverse transcriptase (Promega Corp.). Quantification of gene expression was performed by real‐time PCR using SYBR green fluorescence on a StepOne Real‐Time PCR instrument (Thermo Fisher Sci.). The targeted gene expression levels were calculated using the 2^‐ΔΔCt^ method and were normalized to GAPDH expression.^31^ Primers used in this study are shown here. GAPDH, F, 5′‐ GTCTCCTCTGACTTCAACAGCG, R, 5′‐ ACCACCCTGTTGCTGTAGCCAA; KLF4, F, 5′‐ GAAATTCGCCCGCTCCGATGA, R, 5′‐ CTGTGTGTTTGCGGTAGTGCC; USP11, F, 5′‐ AGGTGTCAGGTCGCATTGAG, R, 5′‐ TGAGAGCCGGTACATCAGGA.

### 2.9 Cell viability and Annexin V/Propidium Iodide (PI) assay

The proliferation was determined using the Cell Counting Kit‐8 (CCK8, Dojindo) cell proliferation assay kit. Briefly, 2 × 10^4^ cells were seeded and pre‐incubated for 24 hours in a 96‐well plate. Medium with different concentrations of FFA was then added, and the cells were incubated for 48 hours. 10 μL of CCK8 solution was added to each well and incubated for 2 hours before measurement with a microplate reader. The absorbance at a wavelength of 450 nm was measured using a microplate reader. In addition, cell viability was also evaluated in HepG2 cells by ATP assay, CellTiter‐Glo kit, following the manufacturer's protocol, and the signal was measured using the GloMax 96 Microplate Luminometer (Promega Corp., WI, USA). All experiments were performed in triplicate. Apoptosis was analysed using an eBioscience Annexin V Apoptosis Detection Kit FITC (Thermo Fisher). Cells were collected by trypsin digestion and adjusted to 1 × 10^6^ cells per 100 μL with 1 × binding buffer, and then, Annexin V‐phycoerythrin (FITC) and propidium iodide (PI) were used to stain cells for 15 minutes in the dark. Flow cytometer instrument (CytoFLEX, Beckman Coulter) was used to analyse the fluorescence of the cells.

### 2.10 Colony forming (clonogenic) assay

HepG2 cells infected with WT or USP11 shRNA particles were selected for 4‐6 days and then were seeded in a 6‐well plate (2500 cell/well). The cells were cultured for 2 weeks, and the medium was refreshed every 2 days. The colonies were fixed and stained with crystal violet. The number of the clones in a given area was counted using ImageJ software.

### 2.11 Publicly available clinical data of HCC and NAFLD

The public RNA‐seq data of HCC patients were obtained from cBioPortal website (https://cbioportal.org). The correlation of mRNA expression between *KLF4* and *USP11* gene was carried out by TCGA PanCancer Atlas data set with 90 hepatocellular carcinoma samples that have over stage 3 of Neoplasm Histologic Grade.^32^ The grade score represents the degree of abnormality of cancer cells, a measure of differentiation and aggressiveness. The range of a set of scores is from grade 1 to grade 4. In addition, the RNA‐seq data of NAFLD patients were obtained from GSE115193 (n = 3),^33^ GSE126848 (n = 15)^34^ and GSE130970 (n = 42, NAFLD activity score >3) in NCBI sequence read archive website (SRA) (https://www.ncbi.nlm.nih.gov/sra). Total 60 samples were selected for the analysis.^35^

### 2.12 RNA expression analysis of RNA‐seq

RNA‐seq samples from patients with HCC or NAFLD were integrated to analyse mRNA expression. SRA toolkit v2.6.2 was performed to download the sequencing data for NAFLD patients from NCBI SRA, and we converted it into fastq format. And the sequencing reads were aligned to the NCBI human genome (GRCh38.p13) using Spliced Transcripts Alignment to a Reference (STAR) 2.7.3a.^36^ The resulting BAM (binary alignment/map) files were processed and normalized using RSEM version 1.3.3 program.^37^ The RSEM normalization method can estimate abundance as gene expression which has recently been developed for accurate estimation. RSEM proposes a statistically directed graph model and uses the expectation‐maximization algorithm to estimate abundances at the gene level considering multiple variables derived from RNA‐seq and transcript data, including library sizes and gene lengths.^38^ In addition, the mRNA expressions of *KLF4* and *USP11* were calculated by RNA‐seq V2 method based on RSEM program. The correlation analysis of mRNA expression of *KLF4* and *USP11* for HCC patients was carried out with ‘ggplot2 version 3.3.0’ and ‘ggpubr version 0.3.0’ package in the statistical environment R‐3.6.3 version.

### 2.13 Statistical analysis

Statistical analysis in this study was performed using GraphPad Prism Software (GraphPad). All data were collected from two or three independent experiments, and the results were expressed as mean  ± SD. One‐way ANOVA, two‐way ANOVA or a two‐tailed Student's *t* test was performed to analyse the statistically significance. *P* values < .05 were considered as significant. *P < .05; **P < .01; ***P < .001, ****P < .0001. In addition, the correlation of mRNA expression was calculated by Pearson correlation.

## 3 RESULTS

### 3.1 USP11 directly interacts with KLF4

To evaluate the underlying molecular mechanisms of KLF4 impaired down‐regulation in tumorigenesis, we used a proteomic approach to identify KLF4‐interacting DUBs in HepG2 cells.^29^ Briefly, whole lysates from Flag‐KLF4 overexpressing HepG2 cells were subjected to immunoprecipitation with anti‐Flag Ab–conjugated agarose beads after extensive pre‐cleaning. The beads were washed and eluted with Flag peptide along with the bounded proteins, which were digested with trypsin and characterized by mass spectrometry. We found several binding USP candidates including USP5, 9X and USP11 (data not shown). Since USP11 has been previously reported as an oncogene in HCC, but its underlying molecular mechanisms in hepatic disease are not entirely understood, we decided to further explore its activity as KLF4‐binding partner.

**FIGURE 1:**
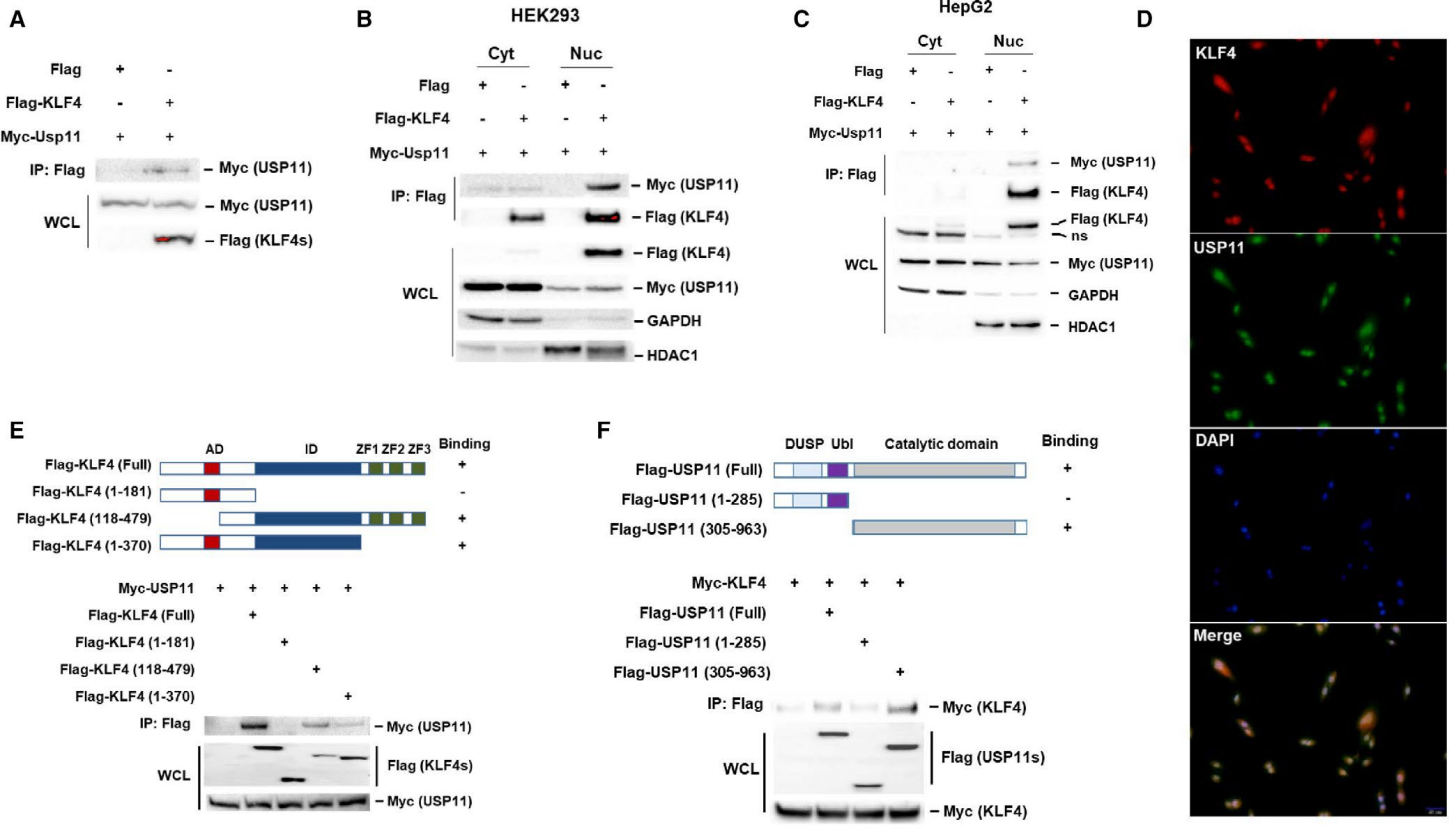
USP11 interacts with KLF4. A, Myc‐USP11 expression plasmid was transfected with or without Flag‐KLF4 expression plasmid into HEK293 cells. KLF4 interaction with USP11 was determined by co‐immunoprecipitation (co‐IP) with anti‐Flag Abs beads followed by immunoblotting with anti‐Myc antibody. The cytosolic and nuclear fractions after cotransfection with Myc‐USP11 and Flag‐KLF4 expression plasmid in HEK293 (B) or HepG2 (C) cells were subjected to IP with anti‐Flag Abs beads and blotted with anti‐Myc antibody. ns: non‐specific. D, Sk‐hep1 cells were fixed and stained with antibodies for KLF4 (red) and USP11 (green). Nuclei were stained with DAPI (blue), and a merged view of the red and green channels within the same field is shown (merge). Scale bar: 50 μm. E, Schematic representation of KLF4 and its mutants, showed in the study. AD, activation domain; ID, inhibitory domain; ZF, zinc finger. Indicated Flag‐KLF4 or deletion mutants with USP11 in transiently transfected HEK293 cells were determined by IP and immunoblotting as described in A. F, Truncated mutants of USP11 were generated and their interaction with KLF4 in transiently transfected HEK293 cells were determined by IP and immunoblotting with indicated antibodies. DUSP, domain present in USPs; Dbl, ubiquitin‐like domain

We started by assessing the interaction between KLF4 and USP11 through co‐immunoprecipitation (co‐IP) analysis in transfected HEK293 cells. Following IP with anti‐Flag beads for Flag‐KLF4, we determined the presence of Myc‐USP11 (Figure 1A). KLF4‐USP11 interaction was mainly found in the nuclear fraction (Figure 1B,C). Immunofluorescence analysis from hepatic adenocarcinoma (Sk‐Hep1) cells showed that the colocalization of both KLF4 and USP11 occurred in the nucleus (Figure 1D). Collectively, these results suggest that USP11 physically interacts with KLF4 in vivo and in vitro. KLF4 has an activation domain (AD) within its amino terminus and contiguous to it has an inhibitory domain (ID), and it has three zinc‐finger DNA‐binding domains at the carboxy terminus. Together, the AD and ID structures allow the binding of other factors including DNA‐binding histone‐modifying enzymes for functional diversity.^5^ To identify the region of KLF4 responsible for the identified interaction with USP11, we generated a series of Flag‐tagged KLF4 deletion mutants. We transfected HEK293 cells with these newly generated Flag‐KLF4 mutants and performed co‐IP and Western blotting analysis similar to the previous experiments, which demonstrated that the KLF4 ID was required for its physical interaction with USP11 (Figure 1E). We also mapped the regions of USP11 that were necessary for the interaction with KLF4. USP11 has a C‐terminal catalytic domain and an N‐terminal regulatory region. Conducting analysis of the impact of serial deletions of USP11 structures showed that deletion of its C‐terminus prevented the interaction with KLF4, whereas deletion of the N‐terminus had only a minor effect on this interaction (Figure 1F). These results demonstrate that the ID domain of KLF4 and the catalytic domain of USP11 are responsible for KLF4‐USP11 interaction.

**FIGURE 2:**
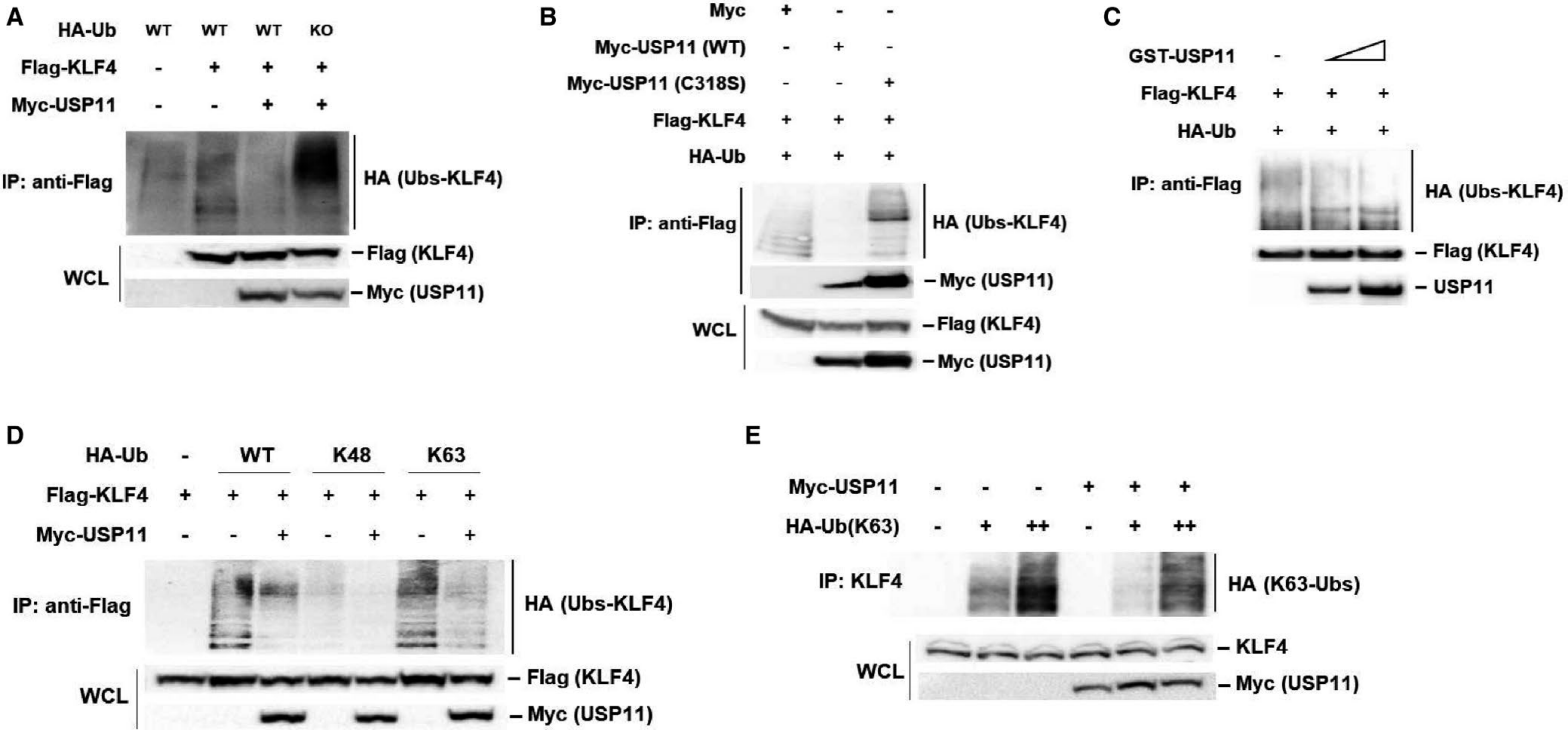
USP11 negatively regulates KLF4 through K63‐linked ubiquitination. A, Flag‐KLF4 and HA‐Ubiquitin (WT‐ or KO‐Ub) plasmids were cotransfected with empty vector or Myc‐USP11 into HEK293 cells. KLF4 ubiquitination was determined by IP of KLF4 with anti‐Flag Abs beads and immunoblotting with anti‐HA antibody. B, Flag‐KLF4 and HA‐Ub plasmids were cotransfected with USP11 (WT or its active‐site mutant C318S). KLF4 protein in lysates of transfected cells was subjected to IP with anti‐Flag Abs beads and KLF4 ubiquitination was analysed by anti‐HA antibody. C, Ubiquitinated Flag‐KLF4 proteins in transient transfected HEK293 cells were pulled down by anti‐Flag Abs beads, followed by incubation with purified GST‐USP11 proteins. KLF4 ubiquitination levels in vitro were determined by Western blotting with anti‐HA antibody. D, KLF4 was cotransfected with HA‐ubiquitin (WT, K48 or K63) and USP11 into HEK293 cells. KLF4 was immunoprecipitated using anti‐Flag Abs beads and immunoblotting with anti‐HA antibody. E, Endogenous KLF4 was isolated using anti‐KLF4 antibody after cotransfection Myc‐USP11 and HA‐K63 Ub plasmids into HepG2 cells and immunoblotting with anti‐HA antibody

Next, we investigated whether USP11 could deubiquitinate KLF4 as a DUB. USP11 and KLF4 were cotransfected into HEK293 cells, along with HA ubiquitin (Ub; wild‐type [WT] or knockout [KO]). The results suggested that coexpression of USP11 and KLF4 significantly prevented the ubiquitination of KLF4 mediated by WT‐Ub, but not from KO‐Ub (Figure 2A). Moreover, mutation of a critical cysteine into serine of USP11 (C318S), which inactivated its catalytic activity,^27^ completely abolished USP11 ability to catalyse KLF4 deubiquitination without affecting the KLF4‐USP11 interaction (Figure 2B). The suppression of KLF4 ubiquitination by the deubiquitinase activity of USP11 was further confirmed by using an in vitro deubiquitination assay (Figure 2C). Incubation of ubiquitinated KLF4 with a purified glutathione S‐transferase (GST)–tagged USP11 inhibited KLF4 ubiquitination. These results indicate that USP11 is a specific deubiquitinase of KLF4 and its functional catalytic domain is required for KLF4 deubiquitination.

### 3.2 USP11 negatively regulates KLF4 through K63‐associated deubiquitination

K48‐linked ubiquitin chains are known to regulate protein levels through proteasomal degradation, whereas K63‐linked ubiquitin chains regulate protein interactions, localization and enzymatic activities, thereby contributing for signal transduction mechanisms related to inflammation, DNA repair and endocytic trafficking as proteasome‐independent processes.^39^ To further explore which type of polyubiquitin chain on KLF4 that was removed by USP11, we transfected HEK293 cells with KLF4 and HA‐tagged ubiquitin mutants in which all lysine, except only one (K48 or K63), were changed to arginine. USP11 was found to suppress increased K63‐linked ubiquitination but not the K48‐dependent mechanism of overexpressed (Figure 2D) or endogenous (Figure 2E) ubiquitination of KLF4. These results suggest that USP11 removes K63‐dependent polyubiquitination in KLF4.

**FIGURE 3:**
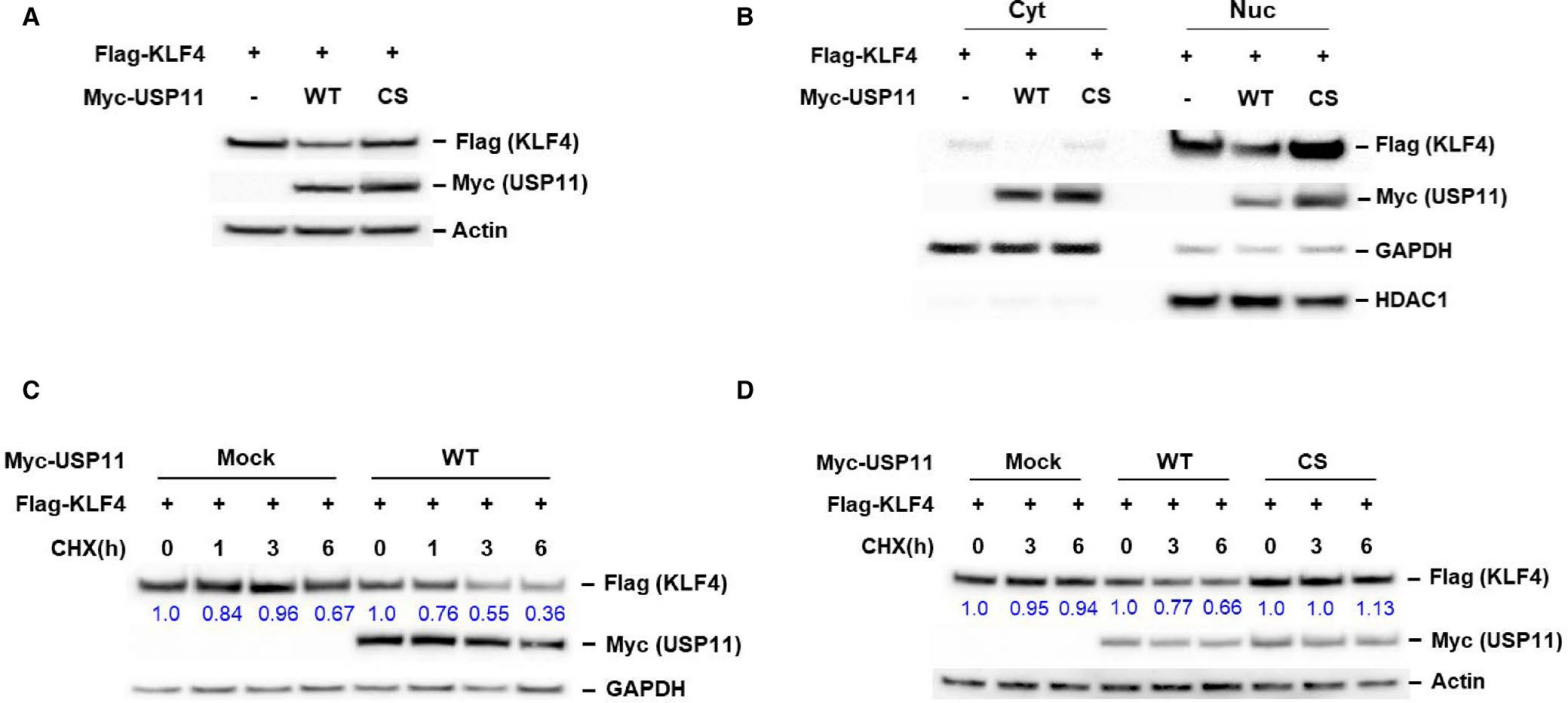
USP11 suppresses KLF4 expression. A, HEK293 cells were transfected with the indicated constructs. Total protein was extracted and subjected to immunoblotting with anti‐Flag, Myc or actin antibody. B, The cytosolic and nuclear fractions were isolated from HEK293 cells cotransfected Flag‐KLF4 with USP11 WT or inactive CS mutant and immunoprecipitated using anti‐Flag Abs beads s and blotted with as described in A. C, KLF4 was cotransfected with empty or USP11 WT plasmids into HEK293 cells. The transfected cells were treated with cycloheximide (CHX, 30 μg/mL) for the indicated time. The protein levels in the treated cells were analysed by Western blotting using anti‐Flag or Myc antibody. Actin or GAPDH was used as a loading control. The band intensities of KLF4 proteins were quantified, and their relative levels are shown. D, Empty, USP11 WT or CS mutant plasmids were transfected with KLF4 into HEK293 cells. KLF4 protein stabilities in the transiently transfected cells were examined as described in C.

To investigate whether USP11 regulated KLF4 levels in a K63‐dependent manner, we evaluated the levels of KLF4 in HEK293 cells transfected with KLF4 plus USP11. Elevated expression of USP11 WT led to decreased KLF4 levels, whereas expression of deubiquitinase a catalytically inactive USP11/CS mutant did not affected KLF4 levels (Figure 3A). Moreover, nuclear KLF4 expression was the most affected fraction rather than the cytoplasmic KLF4 fraction (Figure 3B). These findings suggest that USP11 may regulate KLF4 stability. Next, we cotransfected Flag‐KLF4 with empty, Myc‐USP11 WT or CS mutant plasmids in HEK293 cells treated with cycloheximide (CHX) to inhibit protein biosynthesis. Protein extracts obtained at different time points were analysed to evaluate the stability of the exogenous Flag‐KLF4 protein. USP11 transient overexpression resulted in reduced KLF4 expression and shorter protein half‐life (Figure 3C). In contrast, expression of the deubiquitinase catalytically inactive USP11/CS mutant protected KLF4 from degradation, indicating that KLF4 stabilization requires ubiquitin‐specific peptidase activity by USP11 (Figure 3D). Altogether, these results indicate that USP11 is a KLF4‐specific deubiquitinase that promotes KLF4 degradation.

### 3.3 USP11 mediates cancer cell proliferation and tumorigenesis by promoting KLF4 instability

**FIGURE 4:**
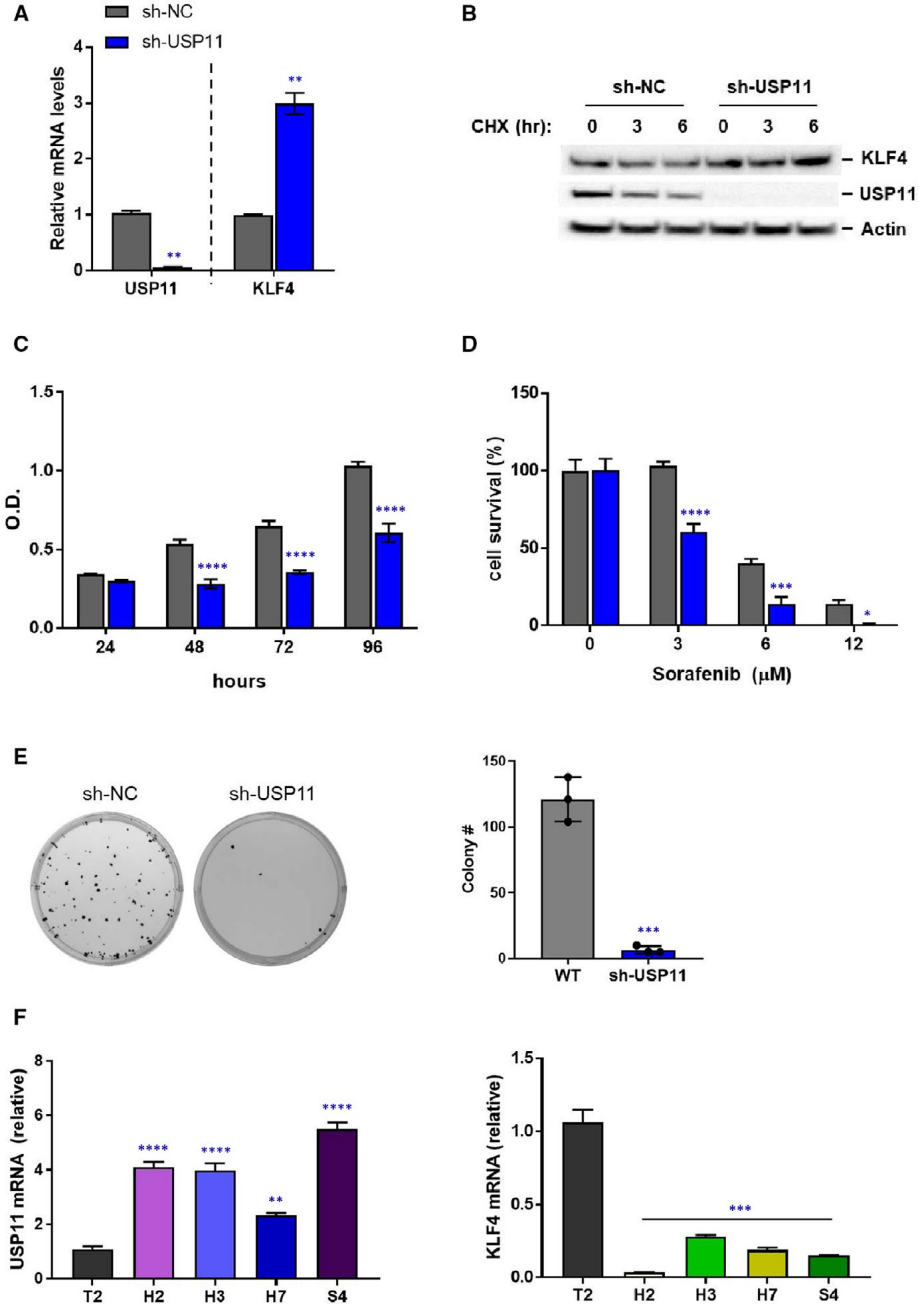
USP11 deletion induces KLF4 expression and inhibits HCC cell proliferation. A, Indicated lentiviral shRNAs (sh‐NC or sh‐USP11) were infected into HepG2 cells. Total RNA was isolated and the levels of *USP11* and *KLF4* were determined by real‐time quantitative PCR. B, HepG2 cells infected with the indicated lentiviral shRNAs were treated with CHX (30 μg/mL) for the indicated time. The protein levels of KLF4, USP11 and actin were analysed by Western blotting. C, HepG2 cells were infected with the NC or USP11 shRNA and cell proliferation was monitored using CCK8 assay at the indicated time points. D, HepG2 cells infected with the indicated lentiviral shRNAs were treated sorafenib (0, 3, 6 or 12 μmol/L) during 24 h. Cell survival was measured using CellTiter‐Glo (Promega). E, Anchorage‐independent colony formation of HepG2 cells stably expressing indicated shRNAs was determined by soft agar assay. Photographs of Petri dishes in a representative experiment and the average number of colonies from three experiment were indicated. F, *KLF4* and *USP11* expression in different liver cells (T2, THLE2; H2, HepG2; H3, Hep3B; H7, Huh7; S4, SNU423) were determined by qRT‐PCR analysis. Relative expression levels are normalized by internal control (GAPDH). Each experiment was repeated 3 times (n = 3). Data represent means ± SD. A and E, two‐tailed Student's *t* test; C and D, two‐way ANOVA; F, one‐way ANOVA, *P < .05; **P < .01; ***P < .001; ****P < .0001

**FIGURE 5:**
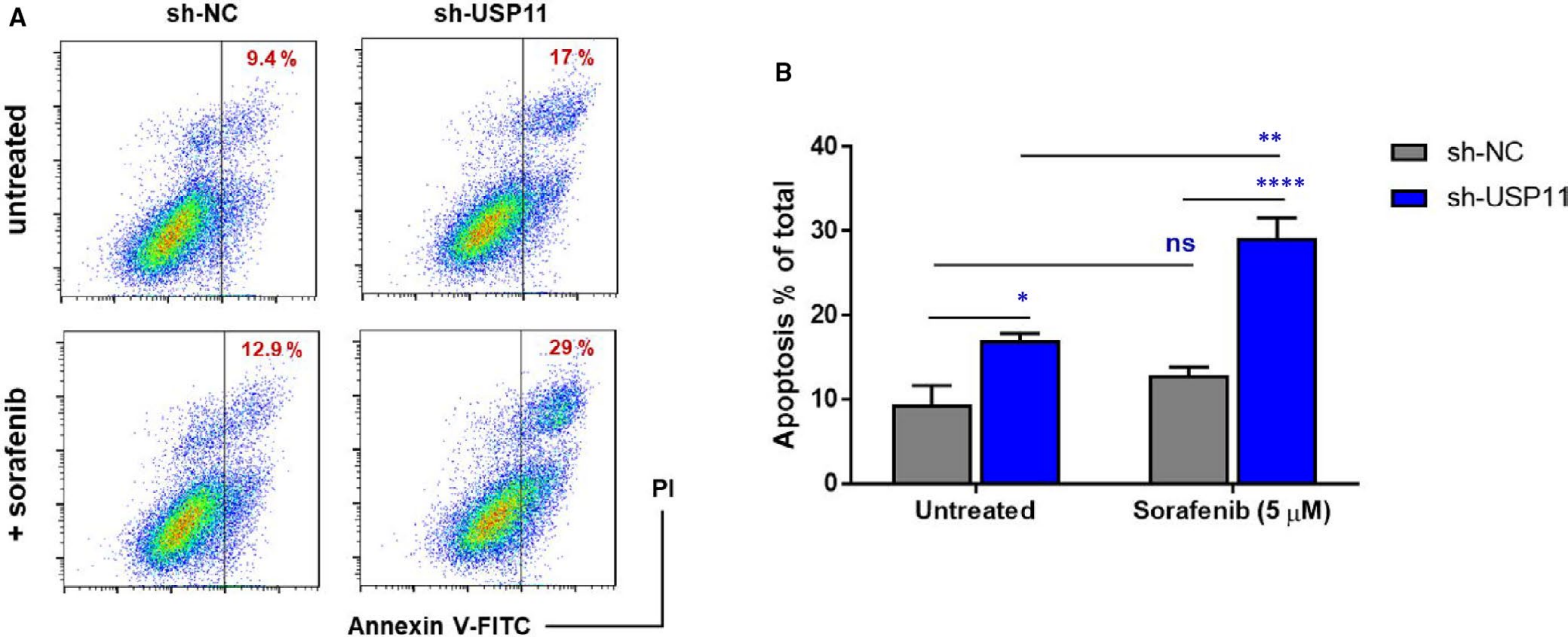
Loss of USP11 induces apoptosis and drug sensitivity in liver cancer cells. HepG2 cells infected with the indicated lentiviral shRNAs were treated with or without sorafenib (5 μmol/L). Apoptosis was determined by flow cytometry analysis using Annexin V and PI staining. In the population of Annexin V–positive cells, PI‐negative (early) or PI‐positive (late and/or necrotic) cells were considered to be apoptotic. Representative flow cytometry data are shown in A. B, Data represent means ± SD (n = 3). *P < .05; **P < .01; ****P < .0001; NS, no significance (two‐way ANOVA). Representative results from 2 independent experiments are shown (n = 2)

Next, we aimed to investigate the function of USP11 in HCC cells. We started by knocking down USP11 expression using specific short hairpin RNAs (shRNAs) in HepG2 cells. Deletion of USP11 led to up‐regulation of *KLF4* expression (Figure 4A) and longer KLF4 half‐life (Figure 4B). These results showed that USP11 may not only regulate KLF4 expression post‐translationally but also at its transcriptional level. Furthermore, down‐regulation of USP11 significantly suppressed HepG2 cell growth (Figure 4C) and chemoresistance (Figure 4D), and clonogenic assay showed that USP11 silencing greatly suppressed the colony‐forming ability of these HCC cells (Figure 4E). We also analysed the relative expression of *USP11* and *KLF4* in some commonly used HCC cells (HepG2, Hep3B, Huh7 and SNU423), as well as in normal hepatocytes (THLE2) (Figure 4F). *USP11* expression was found to be consistently increased in HCC cell lines, whereas KLF4 levels were lower in HCC cells compared with those of normal hepatocytes. To further elucidate the mechanism by which USP11 participates in tumorigenesis, we examined apoptosis by flow cytometry analysis using FITC‐labelled anti‐Annexin V and PI staining (Figure 5). The results suggested that deletion of USP11 could lead to increased apoptosis of HCC cells compared with control cells (expressing USP11) and that USP11‐deleted HepG2 cells were more sensitive to sorafenib treatment. Collectively, these data demonstrate that USP11 down‐regulation sensitizes human HCC cells to apoptosis and suppresses tumour growth by regulating KLF4 stability.

### 3.4 KLF4 level is inversely correlated with USP11 expression in liver disease

**FIGURE 6:**
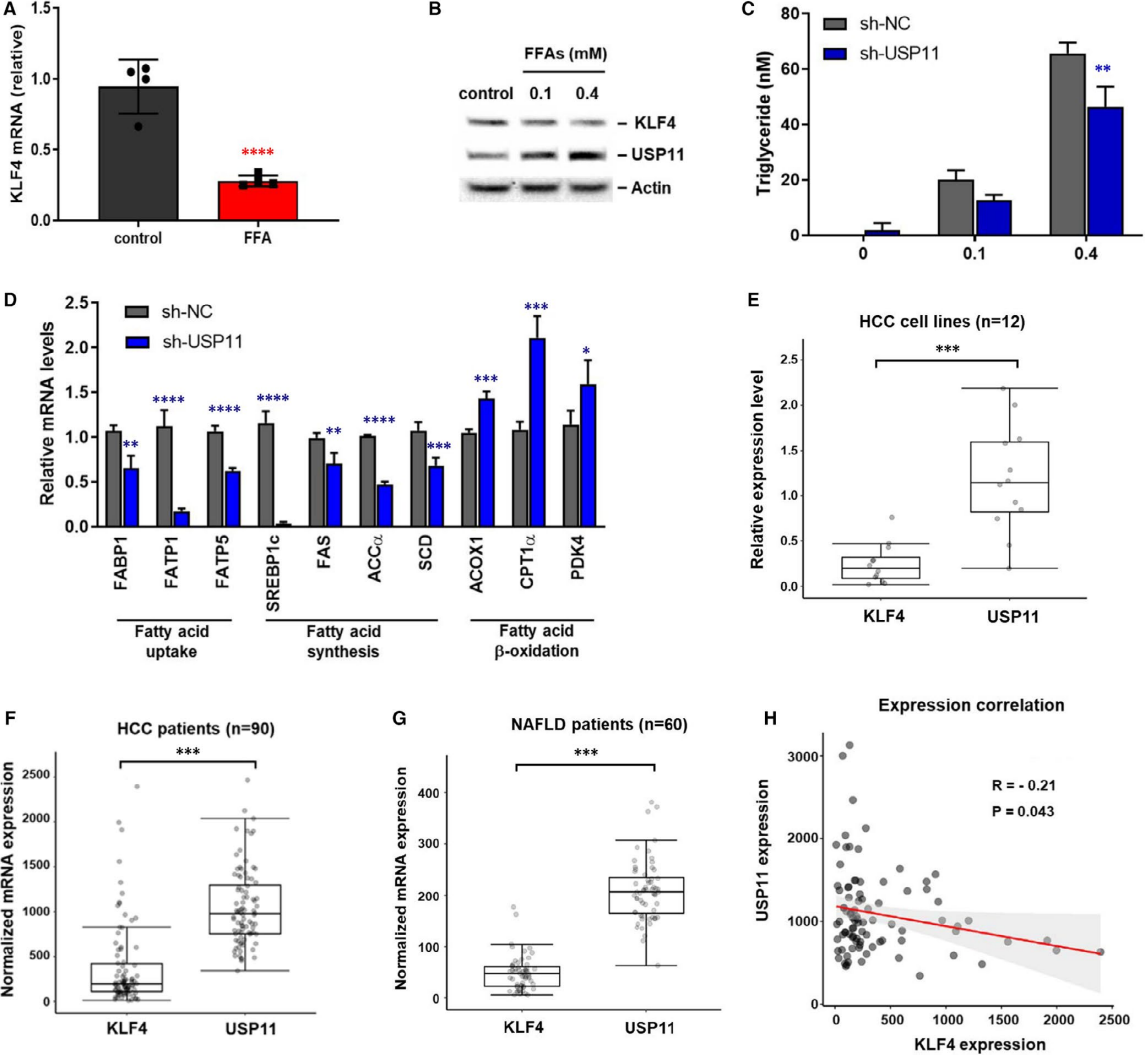
KLF4 level is inversely correlated with USP11 expression in liver disease. A, KLF4 expression levels in free fatty acids (FFA, oleate/palmitate, 2:1 ratio)‐ or vehicle‐treated (control) HepG2 cells. KLF4 mRNA expression level was measured by qRT‐PCR. B, KLF4 and USP11 expression levels in FFA‐treated HepG2 were examined by Western blotting. C, USP11 WT or KO HepG2 cells were treated w/ or w/o FFA. Hepatic steatosis (fatty liver) was determined by intracellular triglycerides (TGs) levels. D, The expression of genes related to fatty acid uptake, synthesis and oxidation in the USP11 WT or KO HepG2 cells after FFA treatment. Representative results from 2 independent experiments are shown (n = 2). E, The mRNA expression levels of *KLF4* and *USP11* from real‐time RT‐PCR in HCC cell lines (HepG2, Hep3B, Huh7, SNU326 and SNU423). The targeted gene expression levels were calculated using the 2^‐∆∆^Ct method and were normalized to GAPDH expression. F, mRNA expression levels of *KLF4* and *USP11* of 90 RNA‐seq data with HCC. The raw data were obtained from https://www.cbioportal.org. G, The RSEM normalized mRNA expression levels of *KLF4* and *USP11* in 60 NAFLD patients by using RNA‐Seq analysis. The sequencing data for NAFLD patients were downloaded from NCBI SRA (https://www.ncbi.nlm.nih.gov/sra). H, The negative correlation between KLF4 and USP11 expression levels was observed in public RNA‐sequencing data from HCC patients. Pearson correlation was −0.21 (*P* < .05). Data represent means ± SD. A and D‐H, two‐tailed Student's *t* test; C, two‐way ANOVA; *P < .05; **P < .01; ***P < .001; ****P < .0001

Non‐alcoholic fatty liver disease (NAFLD) is often related with obesity and metabolic liver disease characterized by steatosis and lipid accumulation in liver cells.^40^ Although hepatic simple steatosis is considered a benign state, it can progress to non‐alcoholic steatohepatitis (NASH), which is a precursor to more serious liver diseases such as cirrhosis and HCC.^41^ To investigate the role of KLF4 in NAFLD pathogenesis, we induced steatosis in vitro by treating HepG2 cells with free fatty acid (FFA)‐bovine serum albumin (BSA) complex (palmitic/oleic acid, 1:2 ratio), or BSA as control, for 24 hours *KLF4* expression was notably decreased in FFA‐treated cells as compared with BSA‐treated cells (Figure 6A), and KLF4 levels were inversely correlated with those of USP11 in FFA‐treated HepG2 cells (Figure 6B). Lipid metabolic imbalance in the liver is a well‐known characteristic of NAFLD. To explore the role of USP11 in hepatic lipid metabolism, we evaluated lipid levels, specifically triglycerides (TGs), and the expression of genes related to fatty acid synthesis, uptake and β‐oxidation, from USP11 WT or KO HepG2 cells. TG content and mRNA levels of fatty acid uptake and synthesis‐related genes were reduced upon USP11 deficiency, whereas mRNA levels of β‐oxidation‐related genes were increased in USP11‐KO HepG2 cells compared with WT cells after FFA treatment (Figure 6C,D). Since we observed that *KLF4* shows the opposite expression trend of *USP11* in HCC cell lines (Figure 6E), we further analysed the correlation between *KLF4* and *USP11* expression from RNA‐sequencing (RNA‐seq) data of HCC or NAFLD clinical cases (Figure 6F,G). The analysis comprised data from 90 patients with HCC (male: 56, female: 34, average age: 59.91) and 60 patients with NAFLD (male: 27, female: 33, average age: 51.29). The overall median *KLF4* expression was of 48 and 195 in the HCC and NAFLD cases, whereas the median of expression value of *USP11* was 207 and 986, respectively, as calculated by a normalization method of RNA‐Seq by Expectation‐Maximization (RSEM) software. The USP11 negatively regulating KLF4 expression in HCC patients was on average augmented by 4.3‐fold (USP11: 207 and KLF4: 48). In addition, USP11 was overexpressed fivefold compared with KLF4 in NAFLD patients (USP11:986 and KLF4:195). The observed differences between *KLF4* and *USP11* expression regarding HCC and NAFLD were statistically significant (*P* < .001). More importantly, we confirmed that *KLF4* levels were negatively correlated with *USP11* expression (Pearson correlation = −0.21, *P* < .05) in data from a public HCC database, further supporting the negative relationship between KLF4 and USP11 in HCC (Figure 6H). These results show that the *KLF4* and *USP11* are reversely express in HCC cells in vitro, as well as in patients.

## 4 DISCUSSION

KLF4 is decreased or absent in HCC cells, and its overexpression has been associated with overall improved survival of patients with HCC. Importantly, KLF4 has been recognized as a HCC prognostic marker.^11^, ^12^ Given its clinical relevance, the study of the molecular mechanisms involved in the down‐regulation of KLF4 in HCC is a growing field; nevertheless, it remains poorly understood. The different roles of KLF4 concerning the different tissue contexts may be determined by expression patterns of its binding partners and the chromatin condition of each cell. KLF4 levels have a high turnover rate, implying that KLF4 is regulated post‐translationally. Among the possible post‐translational modifications, ubiquitination is a dynamic and reversible process that represents an important regulatory mechanism of several cellular functions, including protein degradation, activation, and localization, thereby controlling a wide range of signalling pathways. As ubiquitination has such a broad impact on cell status and fate, its deregulation has been implicated in several human diseases including cancer.^42^ To understand the regulatory mechanisms of KLF4 at the protein level, we used a protein‐complex purification protocol, combined with mass spectrometry to find KLF4 partners. We have identified USP11 as a DUB of KLF4, which can bind directly to the transcription factor. Noteworthy, a recent study has implicated USP11 in HCC metastasis and identified it as a prognostic marker for HCC, although its underlying mechanism in HCC tumorigenesis remains unclear.

Our data show that USP11 down‐regulates KLF4 expression by disrupting K63‐specific polyubiquitin chains. K63‐linked ubiquitination is not preferentially associated with proteasome degradation but it is involved in the regulation of intracellular signalling pathways, such as DNA repair, cell cycle and endocytosis.^39^, ^43^ Interestingly, we also found that KLF4/USP11 interaction occurred mainly in the nucleus. USP11 contains a potential nuclear localization signal (NLS) sequence at 445‐452 amino acids on the C‐terminus that mediates its translocation into the nucleus, which is deleted or inactivated in non‐cancer samples.^44^ In normal cells, USP11 is mainly located in the cytoplasm, whereas it translocates to the nucleus in HCC cells.^28^ This is consistent with our data that show that the catalytic domain of USP11 is critical for its interaction with KLF4 and consequently for KLF4 to exhibit its oncogenic effect. Furthermore, our results also showed that high USP11 levels inhibit KLF4 stability and expression. Given the USP11 negative effect on KLF4 stability, we expected that USP11 could also play an essential oncogenic role in HCC tumorigenesis. Consistent with a previous report, our data revealed that by deleting USP11, it was possible to prevent HCC tumorigenesis, proliferation and chemoresistance and induce cancer cell apoptosis. Moreover, *USP11* and *KLF4* levels were found to be negatively correlated in HCC cells and to have the opposite prognostic trend regarding HCC in the clinical setting, which supports the essential role of USP11 in cancer development. Our results provide a novel mechanism between USP11 and KLF4 that could be a promising strategy for tackling HCC.

HCC progression and prognosis are closely associated with the morphological differentiation index of liver cells, with loss of differentiation markers being often linked to early metastasis. Therefore, differentiation therapy represents promise as an effective strategy for HCC treatment. KLF4 is one of the candidates that could induce HCC differentiation as dysregulation of KLF4 expression promotes poor histological grade of HCC, whereas restoration of KLF4 induces HCC differentiation through HNF4α or HNF6 signals.^45^, ^46^ In contrast, USP11 is related to poor differentiation, invasion and recurrence.^28^ Our data suggest that USP11 is a key molecule that might increase tumorigenicity by disrupting KLF4 stability and consequently modulating HCC differentiation status. Therefore, USP11‐targeting inhibitors may not only sensitize HCC cells to chemotherapeutic agents such as sorafenib, but also induce HCC differentiation. However, further investigation is needed to characterize this potential mechanism. Interestingly, KLF4 and other stemness genes (Oct4, Sox2 and c‐Myc) have proposed as putative targets for HCC therapy because they contributed for maintaining cancer stem cells (CSCs), having strong chemoresistance.^45^, ^47^, ^48^ However, this hypothesis is challenged by current finding, which demonstrated consistent roles of KLF4 as a tumour suppressor and differentiation inducer in HCC cases.^13^

Non‐alcoholic fatty liver disease is characterized by an increased uptake and accumulation of lipids in hepatocytes, and its incidence has increased in recent years because of lifestyle changes.^41^ NAFLD includes a broad spectrum of pathologies, ranging from simple steatosis and NASH to liver cirrhosis. NASH may not only progress to advanced hepatic fibrosis and cirrhosis, even hepatocellular carcinoma, but also significantly increases the risk of other diseases, such as cardiovascular disease and diabetes. We observed that KLF4 expression dramatically decreased while USP11 increased in in vitro steatosis conditions induced by FFA treatment. Consistent with previous results, *KLF4* showed the opposite expression pattern compared with USP11 in both HCC and NAFLD patients, and the expression of *KLF4* was negatively correlated with that of *USP11*. The USP11 negatively regulating KLF4 expression in HCC patients was on average augmented by 4.3‐fold (USP11: 207 and KLF4: 48). In addition, USP11 was overexpressed 5‐fold compared with KLF4 in NAFLD patients (USP11:986 and KLF4:195). These findings suggest that USP11 negatively regulates KLF4 expression in HCC patients.

In summary, this is the first study describing USP11 as a modulator of KLF4 in liver dysfunction. Our functional results provide evidence for the crosstalk between KLF4 and USP11 in hepatic diseases; in particular, they show how USP11 enhances HCC tumorigenesis and steatosis through KLF4 inhibition. Even though no small‐molecule inhibitor for USP11 has been identified to date, our data provide the molecular basis for the development of USP11‐specific drug candidates for treating HCC, including early steatosis.

## CONFLICT OF INTEREST

The authors declare no competing interests.

## AUTHOR CONTRIBUTION

**Heeyoung Yang:** Conceptualization (lead); Data curation (lead); Funding acquisition (lead); Investigation (lead); Project administration (lead); Supervision (lead); Validation (lead); Visualization (lead); Writing‐original draft (lead); Writing‐review & editing (lead). **Daeui Park:** Data curation (supporting); Investigation (supporting); Validation (equal); Visualization (supporting); Writing‐original draft (equal); Writing‐review & editing (equal). **Jeongho Ryu:** Investigation (supporting); Validation (supporting). **Tamina Park:** Investigation (supporting); Visualization (supporting).

## Data Availability

Data available on request from the authors.
